# Total neoadjuvant FOLFIRINOX versus neoadjuvant gemcitabine-based chemoradiotherapy and adjuvant gemcitabine for resectable and borderline resectable pancreatic cancer (PREOPANC-2 trial): study protocol for a nationwide multicenter randomized controlled trial

**DOI:** 10.1186/s12885-021-08031-z

**Published:** 2021-03-23

**Authors:** Q. P. Janssen, J. L. van Dam, B. A. Bonsing, H. Bos, K. P. Bosscha, P. P. L. O. Coene, C. H. J. van Eijck, I. H. J. T. de Hingh, T. M. Karsten, M. B. van der Kolk, G. A. Patijn, M. S. L. Liem, H. C. van Santvoort, O. J. L. Loosveld, J. de Vos-Geelen, B. M. Zonderhuis, M. Y. V. Homs, G. van Tienhoven, M. G. Besselink, J. W. Wilmink, B. Groot Koerkamp, for the Dutch Pancreatic Cancer Group

**Affiliations:** 1 Department of Surgery, Erasmus MC University Medical Center, Rotterdam, The Netherlands; 2 Department of Surgery, Leiden University Medical Center, Leiden, The Netherlands; 3 Department of Medical Oncology, Tjongerschans Hospital, Heerenveen, The Netherlands; 4 Department of Surgery, Jeroen Bosch Hospital, Den Bosch, The Netherlands; 5 Department of Surgery, Maasstad Hospital, Rotterdam, The Netherlands; 6 Department of Surgery, Catharina Hospital, Eindhoven, The Netherlands; 7 Department of Surgery, Onze Lieve Vrouwe Gasthuis, Amsterdam, The Netherlands; 8 Department of Surgery, Radboud University Medical Center, Nijmegen, The Netherlands; 9 Department of Surgery, Isala Hospital, Zwolle, The Netherlands; 10 Department of Surgery, Medisch Spectrum Twente, Enschede, The Netherlands; 11 Department of Surgery, Regional Academic Cancer Center Utrecht, St. Antonius Hospital and University Medical Center Utrecht, Utrecht, The Netherlands; 12 Department of Medical Oncology, Amphia Hospital, Breda, The Netherlands; 13 Department of Internal Medicine, Division of Medical Oncology, GROW - School for Oncology and Developmental Biology, Maastricht UMC+, Maastricht, The Netherlands; 14 Department of Surgery, Cancer Center Amsterdam, Amsterdam UMC, Vrije Universiteit, Amsterdam, The Netherlands; 15 Department of Medical Oncology, Erasmus MC University Medical Center, Rotterdam, The Netherlands; 16 Department of Radiation Oncology, Cancer Center Amsterdam, Amsterdam UMC, University of Amsterdam, Amsterdam, The Netherlands; 17 Department of Surgery, Cancer Center Amsterdam, Amsterdam UMC, University of Amsterdam, Amsterdam, The Netherlands; 18 Department of Medical Oncology, Cancer Center Amsterdam, Amsterdam UMC, University of Amsterdam, Amsterdam, The Netherlands

**Keywords:** Neoadjuvant, FOLFIRINOX, Gemcitabine, Chemoradiotherapy, Localized pancreatic cancer, Intention-to-treat, Randomized controlled trial, Overall survival, Quality of life

## Abstract

**Background:**

Neoadjuvant therapy has several potential advantages over upfront surgery in patients with localized pancreatic cancer; more patients receive systemic treatment, fewer patients undergo futile surgery, and R0 resection rates are higher, thereby possibly improving overall survival (OS). Two recent randomized trials have suggested benefit of neoadjuvant chemoradiotherapy over upfront surgery, both including single-agent chemotherapy regimens. Potentially, the multi-agent FOLFIRINOX regimen (5-fluorouracil with leucovorin, irinotecan, and oxaliplatin) may further improve outcomes in the neoadjuvant setting for localized pancreatic cancer, but randomized studies are needed. The PREOPANC-2 trial investigates whether neoadjuvant FOLFIRINOX improves OS compared with neoadjuvant gemcitabine-based chemoradiotherapy and adjuvant gemcitabine in resectable and borderline resectable pancreatic cancer patients.

**Methods:**

This nationwide multicenter phase III randomized controlled trial includes patients with pathologically confirmed resectable and borderline resectable pancreatic cancer with a WHO performance score of 0 or 1. Resectable pancreatic cancer is defined as no arterial and ≤ 90 degrees venous involvement; borderline resectable pancreatic cancer is defined as ≤90 degrees arterial and ≤ 270 degrees venous involvement without occlusion. Patients receive 8 cycles of neoadjuvant FOLFIRINOX chemotherapy followed by surgery without adjuvant treatment (arm A), or 3 cycles of neoadjuvant gemcitabine with hypofractionated radiotherapy (36 Gy in 15 fractions) during the second cycle, followed by surgery and 4 cycles of adjuvant gemcitabine (arm B). The primary endpoint is OS by intention-to-treat. Secondary endpoints include progression-free survival, quality of life, resection rate, and R0 resection rate. To detect a hazard ratio of 0.70 with 80% power, 252 events are needed. The number of events is expected to be reached after inclusion of 368 eligible patients assuming an accrual period of 3 years and 1.5 years follow-up.

**Discussion:**

The PREOPANC-2 trial directly compares two neoadjuvant regimens for patients with resectable and borderline resectable pancreatic cancer. Our study will provide evidence on the neoadjuvant treatment of choice for patients with resectable and borderline resectable pancreatic cancer.

**Trial registration:**

Primary registry and trial identifying number: EudraCT: 2017–002036-17.

Date of registration: March 6, 2018.

Secondary identifying numbers: The Netherlands National Trial Register – NL7094, NL61961.078.17, MEC-2018-004.

**Supplementary Information:**

The online version contains supplementary material available at 10.1186/s12885-021-08031-z.

## Introduction

Pancreatic ductal adenocarcinoma is often diagnosed at an advanced stage. Only 10–20% of patients present with resectable or borderline resectable pancreatic cancer, for which a potentially curative resection can be performed. Despite surgery, cure remains exceptional, as is demonstrated by a 10-year overall survival (OS) after resection of less than 4% [1]. Most patients die of distant progression rather than local recurrence**.** Apparently, the vast majority of patients with local disease on imaging already have occult metastatic disease. This underlines the importance of systemic therapy.

Upfront surgery with adjuvant gemcitabine has long been the standard of care for patients with resectable pancreatic cancer [2]. Over the past decade, multiple randomized trials have focused on adjuvant therapy, with gradually improving OS [3–5]. Unfortunately, only a subgroup of patients with localized pancreatic cancer receive the intended upfront surgery and adjuvant therapy. First, 10–20% of patients who are scheduled for surgical exploration do not undergo resection, because metastatic or locally unresectable disease is found at surgery that was not anticipated on imaging [6]. An exploratory laparotomy without resection has considerable mortality, morbidity, and a prolonged reduced quality of life. Most of these patients fail to receive palliative chemotherapy [7]. Second, many patients (40–50%) do not recover from a resection sufficiently or in time to tolerate adjuvant chemotherapy [8, 9]. Third, recurrence within 6 months after surgery can occur in up to 50% of patients who do not receive adjuvant chemotherapy [3]. It is unlikely that these patients derived any benefit from surgery. Hence, with upfront surgery, too many patients with the initial diagnosis of resectable or borderline resectable pancreatic cancer undergo futile surgery and too few patients receive systemic chemotherapy, while the majority of patients have occult metastatic disease at presentation.

Neoadjuvant therapy has been proposed to overcome the drawbacks associated with upfront surgery. Single-arm studies on neoadjuvant chemotherapy, with or without radiotherapy, have reported favorable outcomes. A meta-analysis of 38 studies with 3843 patients with resectable and borderline resectable pancreatic cancer found superior OS by intention-to-treat (ITT) (18.8 vs. 14.8 months) and higher R0 resection rates (87% vs. 67%; *p <* 0.001) after neoadjuvant therapy compared with upfront surgery [6]. The addition of radiotherapy to chemotherapy has been suggested to improve R0 resection rate and decrease local recurrence rate, with the potential to improve OS. A recent Korean randomized phase II-III trial was closed early after inclusion of 50 patients because of superior survival with neoadjuvant versus adjuvant gemcitabine-based chemoradiotherapy at interim analysis (21 vs. 12 months, *p* = 0.028) [10]. The Dutch PREOPANC-1 randomized controlled trial (RCT) compared neoadjuvant gemcitabine-based chemoradiotherapy to upfront surgery, both arms followed by adjuvant gemcitabine [11, 12]. Although this study did not meet the primary endpoint of OS by ITT (16.0 vs. 14.3 months, *p =* 0.096), all secondary outcomes found superiority of the neoadjuvant arm: R0 resection rate (71% vs. 40%; *p* < 0.001), disease free survival (8.1 vs. 7.7 months, *p* = 0.032), and locoregional recurrence free interval (not reached vs. 13.4 months, *p* = 0.003).

In 2011, the multi-drug regimen FOLFIRINOX, consisting of 5-fluorouracil with leucovorin, irinotecan, and oxaliplatin, was superior to gemcitabine in patients with metastatic pancreatic cancer (median OS 11.1 vs. 6.8 months, *p* < 0.001) [13]. For locally advanced pancreatic cancer (LAPC), no RCT has been conducted, yet a favorable median OS with FOLFIRINOX of 24 months was found in a patient-level meta-analysis including 315 patients [14]. In comparison, the median OS with gemcitabine for LAPC ranged from 8 to 13 months in previous studies [15]. In the neoadjuvant setting, a patient-level meta-analysis of FOLFIRINOX for borderline resectable pancreatic cancer found a median OS of 22.2 months [16]. In recent years, FOLFIRINOX has become the most commonly used neoadjuvant chemotherapy in observational studies and ongoing phase II trials [17].

Neoadjuvant therapy appears the most appropriate choice for most patients with localized disease. A direct comparison of FOLFIRINOX to gemcitabine-based chemoradiotherapy in the neoadjuvant setting has not yet been performed in a phase III trial. Our primary objective is to determine if total neoadjuvant FOLFIRINOX results in superior OS compared with neoadjuvant gemcitabine-based chemoradiotherapy and adjuvant gemcitabine for patients with resectable and borderline resectable pancreatic cancer.

## Methods

### Design

**Fig. 1:**
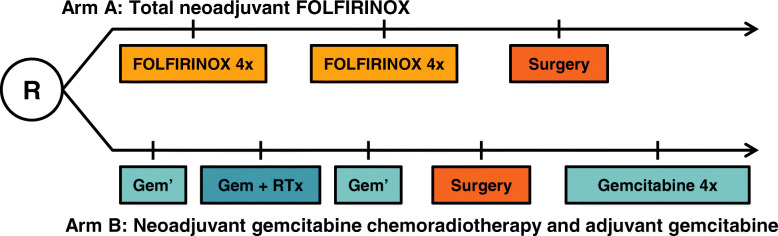
Treatment schedule

The PREOPANC-2 trial is a multicenter randomized phase III superiority trial, initiated by the Dutch Pancreatic Cancer Group (DPCG). A list of all participating centers is added as Supplementary file. Eligible patients are randomly assigned to either receive neoadjuvant FOLFIRINOX followed by surgery without adjuvant treatment (intervention; arm A) or neoadjuvant gemcitabine-based chemoradiotherapy followed by surgery and adjuvant gemcitabine (comparator; arm B) (Fig. 1). Randomization in a 1:1 ratio is performed centrally using a web-based system, with stratification according to center and by resectability status (resectable vs. borderline resectable).

### Study population

Patients are eligible if they have histologically or cytologically confirmed resectable or borderline resectable pancreatic cancer, without distant metastases. Resectability is assessed by a multiphase computed tomography (CT) scan within 4 weeks before randomization. A tumor without arterial (common hepatic artery, superior mesenteric artery, or celiac trunk) involvement and with venous (portal vein and/or superior mesenteric vein) involvement ≤90° is considered resectable; a tumor with arterial involvement ≤90° and/or venous involvement > 90° and ≤ 270° without occlusion is considered borderline resectable. Other inclusion criteria are a World Health Organization (WHO) performance status of 0 or 1, ability to undergo surgery, chemoradiotherapy, and chemotherapy, age ≥ 18 years, adequate bone marrow function (i.e. hemoglobin ≥6 mmol/l; leucocytes ≥3.0 × 10^9^/l; platelet count ≥100 × 10^9^/l), adequate renal function (e-GFR ≥ 50 ml/min), and written informed consent.

Exclusion criteria are prior treatment for pancreatic cancer, comorbidity or previous treatment precluding surgery, chemoradiotherapy, and chemotherapy, and pregnancy. Furthermore, patients are ineligible in case of previous malignancy, unless no evidence of disease and diagnosed more than 3 years before diagnosis of pancreatic cancer, or with a life expectancy of more than 5 years from date of inclusion. A past medical history of non-melanoma skin cancer, pancreatic neuroendocrine tumor (pNET) < 2 cm, and gastrointestinal stromal tumor (GIST) < 2 cm are not exclusion criteria. Lesions on chest CT that are too small to characterize are not considered metastatic disease.

Patients with hyperbilirubinemia may be randomized, but biliary drainage with a metal stent should be performed before start of neoadjuvant therapy if bilirubin is higher than 1.5 times the upper limit of normal.

### Treatment

#### Arm a: total neoadjuvant FOLFIRINOX

Treatment in arm A starts with four cycles of neoadjuvant FOLFIRINOX, followed by a restaging CT-scan. Patients with treatment response or stable disease according to Response Evaluation Criteria in Solid Tumors (RECIST) 1.1 criteria are scheduled for an additional four cycles of neoadjuvant FOLFIRINOX. Restaging CT-scan is repeated and when appropriate followed by surgical exploration with intended resection. No adjuvant chemotherapy is scheduled. Cycles are repeated every 2 weeks (Fig. 1). The dosages are identical to that of the phase III trial (PRODIGE 4/ACCORD 11 trial) for metastatic pancreatic cancer [13]. Starting with a modified regimen is allowed in patients older than 75 years or at the discretion of the treating physician, including withholding of the fluorouracil bolus or dose reduction of irinotecan and oxaliplatin to 80%. Fluorouracil dose should be adjusted or withheld in patients with a (partial) deficiency of the dihydropyrimidine dehydrogenase (DPD) enzyme. Primary prophylaxis with (Peg) Filgrastim (G-CSF) after every cycle of FOLFIRINOX is strongly recommended. Dose adjustments during treatment should be based on the maximum graded toxicity within the previous cycle.

#### Arm B: neoadjuvant gemcitabine-based chemoradiotherapy and adjuvant gemcitabine

Treatment in arm B starts with three cycles of neoadjuvant gemcitabine, adding hypofractionated radiotherapy (36 Gy in 15 fractions during 3 weeks) to the second cycle. Gemcitabine is given weekly for 3 weeks (day 1, 8, and 15) in subsequent 4-week courses, at a dose of 1000 mg per square meter of body-surface area. The first and third cycle are modified to a 3-week course (day 1 and 8). After neoadjuvant therapy, a restaging CT-scan is performed and when appropriate followed by surgical exploration with intended resection. After resection, four cycles of adjuvant gemcitabine are administered (Fig. 1). Adjuvant chemotherapy should start after the patient has recovered from surgery, but no later than 12 weeks after surgery.

#### Surgery: both groups

Patients are eligible for a surgical exploration if they have non-metastatic resectable or borderline resectable disease on restaging CT-scan of the chest and abdomen. Surgery is performed 3 to 6 weeks after completion of chemotherapy. Surgery starts with a staging laparoscopy (during the same surgical procedure), followed by the standard surgical exploration and resection depending on the location of the tumor. Postoperative complications are defined according to the Clavien-Dindo classification and definitions of post-pancreatic surgery complications (i.e. pancreatic fistula, delayed gastric emptying, and bleeding) according to the International Study Group of Pancreatic Surgery (ISGPS), recorded until 90 days after surgery [18–21]. If chemotherapy is discontinued because of toxicity or in case of local progression at restaging, patients may also proceed to surgical exploration. Patients with distant metastasis or unresectable disease at restaging or surgery continue with standard palliative care according to the national guideline.

### Outcomes

The primary endpoint is OS by intention-to-treat, calculated from date of randomization. Secondary endpoints include progression-free survival, locoregional progression-free interval, distant metastases-free interval, resection rate, R0 resection rate, chemotherapy start rate, chemotherapy completion rate, toxicity, postoperative complications, radiologic response, tumor marker response (serum carbohydrate antigen 19–9 (CA 19–9) and carcinoembryonic antigen (CEA)), pathologic response, and quality of life.

Progression-free survival is defined as survival without any locoregional progressive disease, distant metastases, recurrence, or secondary pancreatic cancer, calculated from the date of randomization. Death from any cause is also considered an event for this endpoint. Patients alive and free of these events will be censored at the last follow-up. For locoregional progression-free interval and distant metastases-free interval, only progression is considered an event and patients are censored at death or at the date of last follow-up for patients alive and free of these events. Resection is considered R0 if the distance between the inked margin and tumor cells is ≥1 mm [22]. Radiologic response is defined according to RECIST criteria version 1.1 comparing pre-randomization and restaging imaging after 4 and 8 cycles of FOLFIRINOX (arm A) or after chemoradiotherapy (arm B). These time points are also used to assess tumor marker response. Pathologic response is defined using the modified 3-tier histologic tumor regression grading (HTRG) scheme [23].

### Quality of life

Quality of life is assessed using questionnaires at multiple time points throughout the study and during follow-up: every 3 months in the first year, every 6 months in the second year, and annually in year 3 to 5.

### Follow-up

After randomization, follow-up takes place every 3 months during the first 2 years and every 6 months during year 3 to 5. Follow-up CT-scans of the chest and abdomen combined with tumor marker analysis (CA 19–9 and CEA) take place at 6, 12, 18, and 24 months from randomization and yearly thereafter, until disease recurrence or up to a maximum of 5 years after randomization in patients without recurrence.

### Data collection and management

The web-based software tool ALEA (FormsVision BV, Abcoude, The Netherlands) is used for randomization, clinical data collection, and central data management. Data management is coordinated by the Clinical Trial Center Rotterdam and data collection is performed by The Netherlands Comprehensive Cancer Organization (Integraal Kankercentrum Nederland). Data entry is done according to study specific data entry guidelines, promoting a uniform and standardized way of data entry and providing procedures for exceptions (i.e. missing values, unknowns). Data managers are trained in using the ALEA electronic case report form system prior to data entry start.

### Monitoring

Throughout the trial, a trained, qualified, and independent monitor will periodically visit each participating center in order to randomly check compliance with the protocol, compliance with in- and exclusion criteria, proper implementation, conduct of Informed Consent procedures, Source Data Verification (i.e. crosscheck data in ALEA with patient dossier and vice versa), and reporting of serious adverse events (SAEs). Adverse events are graded using the Common Terminology Criteria for Adverse Events (CTCAE) version 4.0.3 [24]. SAE’s defined as adverse events grade 3, 4, or 5 are collected. Suspected Unexpected Serious Adverse Reactions (SUSARs) are reported to the Competent Authority and Ethics Committee according to national regulation. In addition to the expedited reporting of SUSARs, the sponsor submits a safety report to the Competent Authority and Ethics Committee once a year during the clinical trial. An independent Data Safety Monitoring Board (DSMB) monitors the safety of the trial subjects by qualitative analyses of feasibility, accrual rate, mortality, and SAE’S after 50 and 100 patients have completed treatment.

### Statistical analysis

Sample size calculation was performed for the primary endpoint of OS. The median OS of 17 months for the chemoradiotherapy arm of the PREOPANC-1 trial (preliminary results, 149/176 events) was used as estimate for the comparator arm [25]. In order to detect a hazard ratio (HR) of 0.70 with 80% power (2-sided significance level alpha = 0.05), a total of 252 events (deaths) need to be observed. This HR translates into a median OS of about 24 months in the intervention arm, which is consistent with a large patient-level meta-analysis on neoadjuvant FOLFIRINOX treatment for borderline resectable pancreatic cancer [16]. The number of events is expected to be reached after inclusion of 368 eligible patients assuming an accrual rate of 10 patients per month with an accrual period of 3 years and an additional follow up of 1.5 years after the last patient has been randomized. Dropouts were rare in PREOPANC-1 and are therefore not accounted for. No interim analysis for the primary outcome is planned.

All main analyses will be performed by intention-to-treat. Cox regression analysis will be performed to calculate the hazard ratio and corresponding 95% confidence interval. Kaplan-Meier method will be used to estimate OS probabilities at appropriate time points, using the Greenwood estimate to construct corresponding 95% confidence intervals (CIs). A *p*-value of 0.05 is considered statistically significant.

Prespecified subgroup analyses include: patients that received at least one cycle of neoadjuvant treatment, patients that underwent a resection, patients that underwent an R0 resection, patients that completed all scheduled treatment, for the subgroups resectable and borderline resectable pancreatic cancer, patients younger vs. older than 65 years, patients with high and low CA 19–9, and patients with performance score 0 vs. 1.

## Discussion

Herein, we describe the protocol of the PREOPANC-2 trial, a multicenter randomized phase III trial conducted by the Dutch Pancreatic Cancer Group in the Netherlands, which was designed to compare the efficacy of two neoadjuvant treatment strategies for patients with resectable and borderline resectable pancreatic cancer. This study builds upon the results of the previously conducted PREOPANC-1 trial [11]. If the PREOPANC-2 trial demonstrates superior OS for patients receiving neoadjuvant FOLFIRINOX, this treatment should be implemented as neoadjuvant treatment of choice for patients with resectable and borderline resectable pancreatic cancer.

Based on the available evidence, we believe that neoadjuvant therapy is the best approach for the majority of patients with both resectable and borderline resectable pancreatic cancer. This paradigm shift was confirmed by a recently published study by Cloyd and colleagues [26]. This meta-analysis of six RCTs comparing neoadjuvant treatment to upfront surgery for resectable and borderline resectable pancreatic cancer patients showed that neoadjuvant treatment significantly improved OS by intention-to-treat compared with upfront surgery (HR 0.73, 95% CI: 0.61–0.86). The pooled HR remained in favor of neoadjuvant treatment in all subgroup analyses, thus independent on anatomic classification (resectable: HR 0.73, 95% CI: 0.59–0.91; borderline resectable: HR 0.51, 95% CI: 0.28–0.93) or neoadjuvant treatment type (chemoradiotherapy: HR 0.77, 95% CI: 0.61–0.98; chemotherapy alone: HR 0.68, 95% CI: 0.54–0.87) In addition, neoadjuvant treatment increased the likelihood of an R0 resection (RR 1.51, 95% CI: 1.18–1.93).

Since the design of the PREOPANC-2 trial, two RCTs showed superiority of gemcitabine combined with capecitabine (ESPAC-4 trial) and modified (m) FOLFIRINOX (PRODIGE 24/CCTG PA.6 trial) when compared to gemcitabine monotherapy in the adjuvant setting [5, 27]. Based on these studies, both mFOLFIRINOX and gemcitabine with capecitabine have become preferred regimens in the adjuvant setting for patients with adequate performance status. It remains unclear what the best adjuvant regimen is after neoadjuvant chemoradiotherapy and resection.

## Trial status

The PREOPANC-2 trial is a nationwide multicenter randomized phase III trial, conducted in 15 centers that provide multidisciplinary treatment for pancreatic cancer throughout the Netherlands. The study opened for accrual on June 5th, 2018. At the time of submission of this paper, all centers were actively recruiting and treating patients. A total of 294 patients were included in the trial on September 1st, 2020.

## Supplementary Information

**Additional file 1: Supplementary file PREOPANC-2 trial.**

## Data Availability

Results will be communicated via presentations at international conferences and via publications in peer reviewed journals. Study protocol is available at the website of the Dutch Pancreatic Cancer Group (www.dpcg.nl). Data generated or analysed during the current study will be available from the corresponding author on reasonable request.

## Abbreviations

CA 19–9: Carbohydrate antigen 19–9
CEA: Carcinoembryonic antigen
CI: Confidence interval
CT: Computed tomography
DPCG: Dutch Pancreatic Cancer Group
DPD: Dihydropyrimidine dehydrogenase
DSMB: Data Safety Monitoring Board
eGFR: Estimated glomerular filtration rate
FOLFIRINOX: 5-fluorouracil with leucovorin, irinotecan, and oxaliplatin
GCP: Good Clinical Practice
G-CSF: Granulocyte colony stimulating factor
GIST: Gastrointestinal stromal tumor
HR: Hazard ratio
HTRG: Histologic tumor regression grading
ISGPS: International Study Group of Pancreatic Surgery
ITT: Intention-to-treat
LAPC: Locally advanced pancreatic cancer
OS: Overall Survival
pNET: Pancreatic neuroendocrine tumor
RCT: Randomized controlled trial
RECIST: Response evaluation criteria in solid tumors
SAE: Serious adverse event
SUSAR: Suspected Unexpected Serious Adverse Reactions
WHO: World Health Organization
