# Supplementary material for: Total neoadjuvant FOLFIRINOX versus neoadjuvant gemcitabine-based chemoradiotherapy and adjuvant gemcitabine for resectable and borderline resectable pancreatic cancer (PREOPANC-2 trial): study protocol for a nationwide multicenter randomized controlled trial
Source: BMC Cancer. 2021 Mar 23;21:300. doi: 10.1186/s12885-021-08031-z (PMC7989075; doi:10.1186/s12885-021-08031-z)
Supplement: Supplementary file 1 — Additional file 1: Supplementary file PREOPANC-2 trial. [file 12885_2021_8031_MOESM1_ESM.docx]

**Supplementary file PREOPANC-2 trial**

**List of participating centers:**

Amphia Hospital, Breda, The Netherlands

Amsterdam UMC, University of Amsterdam, Amsterdam, The Netherlands

Amsterdam UMC, VU University, Amsterdam, The Netherlands

Catharina Hospital, Eindhoven, The Netherlands

Erasmus MC University Medical Center, Rotterdam, The Netherlands

Isala Hospital, Zwolle, The Netherlands

Jeroen Bosch Hospital, Den Bosch, The Netherlands

Leiden University Medical Center, Leiden, The Netherlands

Maasstad Hospital, Rotterdam, The Netherlands

Maastricht UMC+, Maastricht, The Netherlands

Medisch Spectrum Twente, Enschede, The Netherlands

Onze Lieve Vrouwe Gasthuis, Amsterdam, The Netherlands

Radboud University Medical Center, Nijmegen, The Netherlands

Regional Academic Cancer Center Utrecht, St. Antonius Hospital and University Medical Center Utrecht, The Netherlands

Tjongerschans Hospital, Heerenveen, The Netherlands

**List of affiliated centers:**

Elisabeth TweeSteden Hospital, Tilburg, The Netherlands

Meander Medical Center, Amersfoort, The Netherlands

NorthWest Clinics, Alkmaar, The Netherlands

Reinier de Graaf Hospital, Delft, The Netherlands
